# Difference in Striae Periodicity of Heilongjiang and Singaporean Chinese Teeth

**DOI:** 10.3389/fphys.2017.00442

**Published:** 2017-06-29

**Authors:** Sharon H. X. Tan, Yu Fan Sim, Chin-Ying S. Hsu

**Affiliations:** ^1^Ministry of Health HoldingsSingapore, Singapore; ^2^Faculty of Dentistry, National University Health System, National University of SingaporeSingapore, Singapore; ^3^Department of Dentistry, Faculty of Dentistry, National University Health System, National University of SingaporeSingapore, Singapore

**Keywords:** striae periodicity, striae of Retzius, cross-striations, circaseptan interval, enamel

## Abstract

Striae periodicity refers to the number of cross-striations between successive lines of Retzius in tooth enamel. A regular time dependency of striae periodicity, known as the circaseptan interval, has been proposed. Previous studies on striae periodicity have been carried out on both modern and early humans given its potential applications in forensic age estimations and anthropology. Nevertheless, research comparing striae periodicities across gender groups and populations in different geographical locations, particularly in Asia, is lacking. In this study, we compared the striae periodicities of Heilongjiang and Singaporean Chinese, as well as that of Singaporean Chinese males and females. Results showed that while the median striae periodicity counts of Heilongjiang Chinese and Singaporean Chinese teeth are both 7, Heilongjiang Chinese tend to have lower striae periodicity counts than Singaporean Chinese (*p* < 0.01). No significant gender difference was observed between the median striae periodicity of Singaporean Chinese Female and Singaporean Chinese Male teeth (*p* = 0.511). We concluded that the median striae periodicity may statistically differ with geographical location, but not gender, provided that ethnicity and geographical location are held constant. Further studies are required to examine the causes for variation in striae periodicities between geographical locations, as well as to verify the other bio-environmental determinants of striae periodicity.

## Introduction

**Figure 1:**
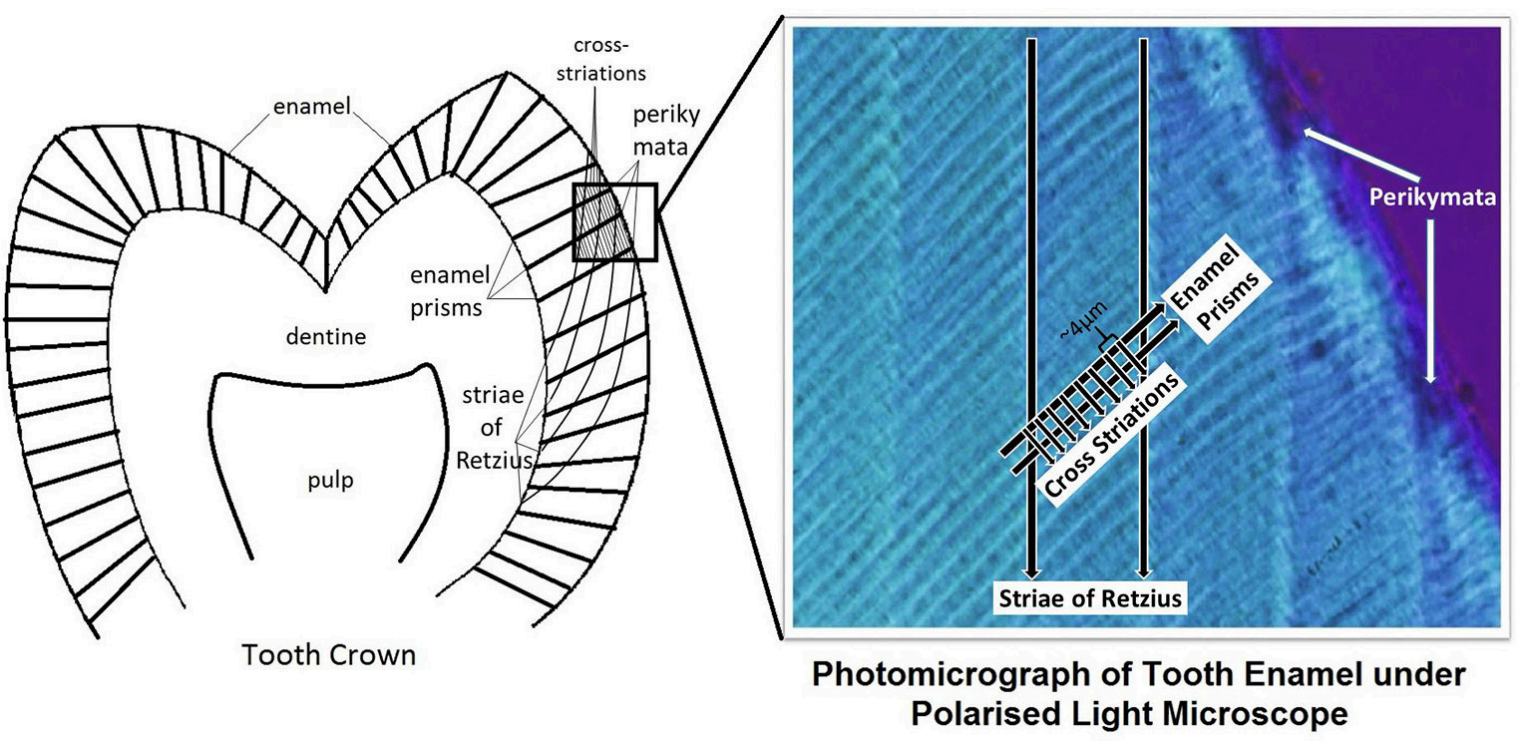
Schematic diagram of tooth.

Enamel is the outermost layer of the anatomical crown of the human tooth. It is formed via amelogenesis, a process that comprises of a presecretory stage, secretory stage, transition stage, maturation stage and post-maturation stage (Berkovitz et al., 2009). Incremental lines have been observed in enamel. Cross-striations are seen under light microscope as small transverse lines that run perpendicular to the long axis of enamel prism, and are approximately 4 μm apart (Risnes, 1986). Results from direct experimental studies indicate that cross-striations correlate with a circadian rhythm of ameloblast secretion activity (Schour and Poncher, 1937). In contrast, Striae of Retzius are a manifestation of a long-period incremental growth. Under transmitted light microscope, they appear as dark lines that run at an oblique angle to enamel prisms and cross-striations (Smith, 2006), and emerge on the enamel surface as grooves called perikymata (Figure 1). Striae of Retzius occur due to the slowing down of ameloblastic activity at regular intervals (Bromage et al., 2009). Striae periodicity refers to the number of daily increments, represented by cross-striations, between two adjacent striae of Retzius. By counting the number of cross striations between striae of Retzius (Berkovitz et al., 2009), by calculation based on a division of the distance between adjacent striae by the average cross striation repeat interval (FitzGerald, 1998), or by estimation (Reid and Ferrell, 2006), striae periodicity has been determined (Supplementary Table 1). A potential explanation for the regular periodicity of striae of Retzius is chronobiology, or the adaptation of cyclic phenomena in living organisms to solar and lunar related rhythms (DeCoursey et al., 2009). Seasons, earth magnetism, solar flares and sunspots appear to have a correlation with human heart rate and melatonin cycles (Cornelissen et al., 2010), and studies suggest that genetically-encoded physiologic circaseptan (7 day) rhythms in humans were evolutionarily adapted to heliogeomagnetic environmental circaseptans (Cornelissen et al., 1998). Although the exact reason remains uncertain, other causes of the regular striae periodicity in enamel could include biologic rhythms controlled by the suprachiasmatic nucleus (Hastings, 1997b), and hormonal controls. Rats with lesions of the suprachiasmatic nucleus have shown disruptions in dentinal incremental lines, while growth and parathyroid hormones known to affect odontoblasts are thought to be responsible for the circadian rhythm of dentin increments (Ohtsuka-Isoya et al., 2001). In addition, melatonin levels increase at night and decrease in the day (Hastings, 1997a), which has been found to correspond with darker stained layers in dentine at night and lighter stained layers in dentine incremental lines of Sprague-Dawley rats (Mishimaa et al., 2012). These suggest the influence of biologic rhythms and hormones on periodic markings in teeth, which may include the Striae of Retzius.

Extensive studies have shown no difference between striae periodicities in different parts of a single tooth (Fukuhara, 1959; Beynon, 1992; FitzGerald, 1998; Mahoney, 2008, 2012). Within an individual, periodicity values are also consistent regardless of the tooth type, or the jaw arch from which the tooth is taken (FitzGerald, 1998; Reid et al., 1998; Mahoney, 2012). Nevertheless, inter-individual variations in striae periodicities have been noted. Proposed factors affecting striae periodicity include gender, ethnicity, body mass and metabolic rate differences (Schwartz et al., 2001; Bromage et al., 2009). However, body mass differences have been said to account more for inter-species or inter-taxa variations in striae periodicity (Schwartz et al., 2001; Smith et al., 2003). Other potential influences include temperature, diet, pH and fluoride levels that affect amelogenesis (Humphrey et al., 2008; Bronckers et al., 2009; Lacruz et al., 2010), although there is currently no direct evidence for these.

With regards to gender effects, a study by Smith et al. (2007) showed inconsistent developmental differences between males and females. While females were found to have a significantly higher periodicity in the South African sample, no significant gender difference was found in the North American sample. No statistical differences in striae periodicity were noted between male and female homo sapiens, despite a faster daily enamel secretion rate in female hominoids (Schwartz et al., 2001). The overall reported range of mean striae periodicity of modern humans is from 6 to 12.3 (FitzGerald, 1998; Reid and Dean, 2006; Supplementary Table 1). The mean striae periodicity values of South African, Northern England, and Northern American populations has been found to be 8.6, 8.1, and 7.9 respectively with South Africans demonstrating a statistically higher mean periodicity than other continental groups (Smith et al., 2007). On the other hand, the mean striae periodicity of early Homo, Australopithecines, and Medieval Danish has been cited as 8, 7 (Lacruz et al., 2006) and 8.5 (Reid and Ferrell, 2006) respectively. Modal periodicity values of early humans range from 7 to 9 (Lacruz et al., 2006).

Given the lack of research comparing striae periodicities across gender groups and populations in different geographical locations, particularly in the Asian context, we sought to examine and compare the striae periodicities of Heilongjiang (China) Chinese and Singaporean Chinese, as well as that of Singaporean males and females in this study.

## Materials and methods

A total of 35 non-carious and non-restored permanent teeth from 35 patients were collected from various dental clinics in Singapore. Another 35 teeth were conveniently collected from various hospitals and clinics in Heilongjiang, China. The teeth were indicated for extraction by dentists for patients' needs. Institution Research Board approval (B-14-004E) was obtained before the start of the study.

Teeth samples were washed in distilled water immediately following extraction, and cleaned thoroughly to remove debris and soft tissues. They were then stored in saline (0.5 Eq/L) in separate labeled containers tagged with a biohazard sign.

A Buehler Isomet Low Speed Saw with a cutting diamond-wafering blade was used to section the teeth longitudinally from cusp tip to cemento-enamel junction with section planes oriented buccolingually and centered through the tips of cusps and the underlying dentine horns. The blade speed was kept at 4 (Marks et al., 1996). Sections were then carefully removed using a cutter blade to obtain two sections per tooth.

Following, the sections were hand ground using a graded series of gradually finer grit Buehler Met-II grinding pads (P800, P1000, P1200, P2500, P4000) with silicon carbide abrasive, on a Buehler Phoenix Beta Grinding/Polishing Machine, until a thickness of 80–100 μm (Reid et al., 1998) was attained and verified with vernier calipers. Each section was then rinsed with distilled water and air-dried for 24 h to remove smear layers and contaminants from the surface of the section. Sections were then mounted onto a glass slide.

Ground sections were examined under an Olympus BX 51 polarized light microscope at 100X magnification. Prior to viewing, a drop of quinoline solution was applied onto each specimen to reduce the optical mismatch of reflective index at enamel-air and enamel-water interfaces (Brodbelt et al., 1981). The outer enamel between lateral and cervical enamel, where striae of Retzius and cross striations are generally most prominent (Lacruz et al., 2006), was first examined. If the striae of Retzius or cross-striations were unclear, the opposite buccal or lingual site was examined. If no results were yielded, adjacent sites were chosen. The section was excluded if enamel imbrication lines could not be clearly visualized at any site of the section. Digital images were produced using a digital microscope camera (Olympus DP25), and captured using imaging software (Olympus Cell D).

Striae periodicity was measured by direct counting of the number of cross-striations between two adjacent striae on captured photomicrographs, by three independent observers. The number of cross-striations was thus measured in whole integers. The median of the striae periodicity values for each tooth based on the six readings from the two sections (or three readings if one section was excluded) was then determined.

To reduce the inaccuracy of results, care was taken to distinguish specimen and optical artefactsfrom cross-striations and striae of Retzius (Mann et al., 1990). All striae counted were traced to their emergence as perikymata (Antoine and Dean, 2009). Only sections with good image quality, without excessive overlap between enamel prisms, and with consistent striae periodicity counts, were included.

All statistical analyses were carried out using STATA Version 14 (StataCrop. 2015. Stata Statistical Software: Release 14. College Station, TX: StataCorp LP). The extent to which each of the three observers gave consistent striae periodicity counts of the same sample (intra-observer reliability) was assessed. Using a random number generator, five sections were selected and an independent repeat count was done by each observer. The repeat striae periodicity counts were then compared with periodicity counts of the corresponding sections recorded in our results, using single-measure intraclass correlation coefficient (ICC) with a one-way random model. To examine for the extent to which the three observers give consistent striae periodicity counts of the same sample (inter-observer reliability), the corresponding readings for the three observers for all sections were assessed using average-measure ICC with a two-way mixed effect model. ICC reflects the degree of agreement between observations by studying the variation of observations from the same sample. The Kolmogorov-Smirnov test was employed for a test of normality, and a non-parametric approach was applied in the event of violation of the normality assumption. The Mann-Whitney U-test was applied to compare the striae periodicities of Heilongjiang Chinese and Singaporean Chinese, as well as that of Singaporean males and females. The significance level for tests was set at 5%.

## Results

Out of the 35 Singaporean Chinese teeth, one was excluded as cross-striations and Retzius lines could not be clearly determined from the two sections of the tooth. Seven Heilongjiang Chinese teeth and four Singaporean Chinese teeth had only one section (instead of two) that was determined to be diagnostically acceptable.

Striae periodicity values of the 34 remaining extracted tooth samples from the Singapore population (Supplementary Table 3) and the 35 tooth samples from the Heilongjiang population (Supplementary Table 2) were finalized and analyzed. Within the Singaporean population, the data was further segregated into two groups: striae periodicity values of the 18 teeth from Chinese Male Singaporean residents, and that of the 16 teeth from Chinese Female Singaporean residents.

To first establish that the observations from the two sections from each tooth were similar, a test for homogeneity between the two sections was performed using the Wilcoxon Sign Rank test. No statistically significant differences were observed between the striae periodicity counts of two sections from each tooth (*p* = 0.593). The ICC of average measures on absolute agreement to measure inter-observer reliability was reported at 0.92. On the other hand, the ICC to measure intra-observer reliability for Observer A, Observer B, and Observer C was 0.71, 0.72, and 0.71 respectively. In view of the homogeneity between the sections from each tooth and high degree of inter-observer reliability on striae periodicity counts, the striae periodicity count of each tooth was summarized using the median reading across sections and observers.

**Figure 2:**
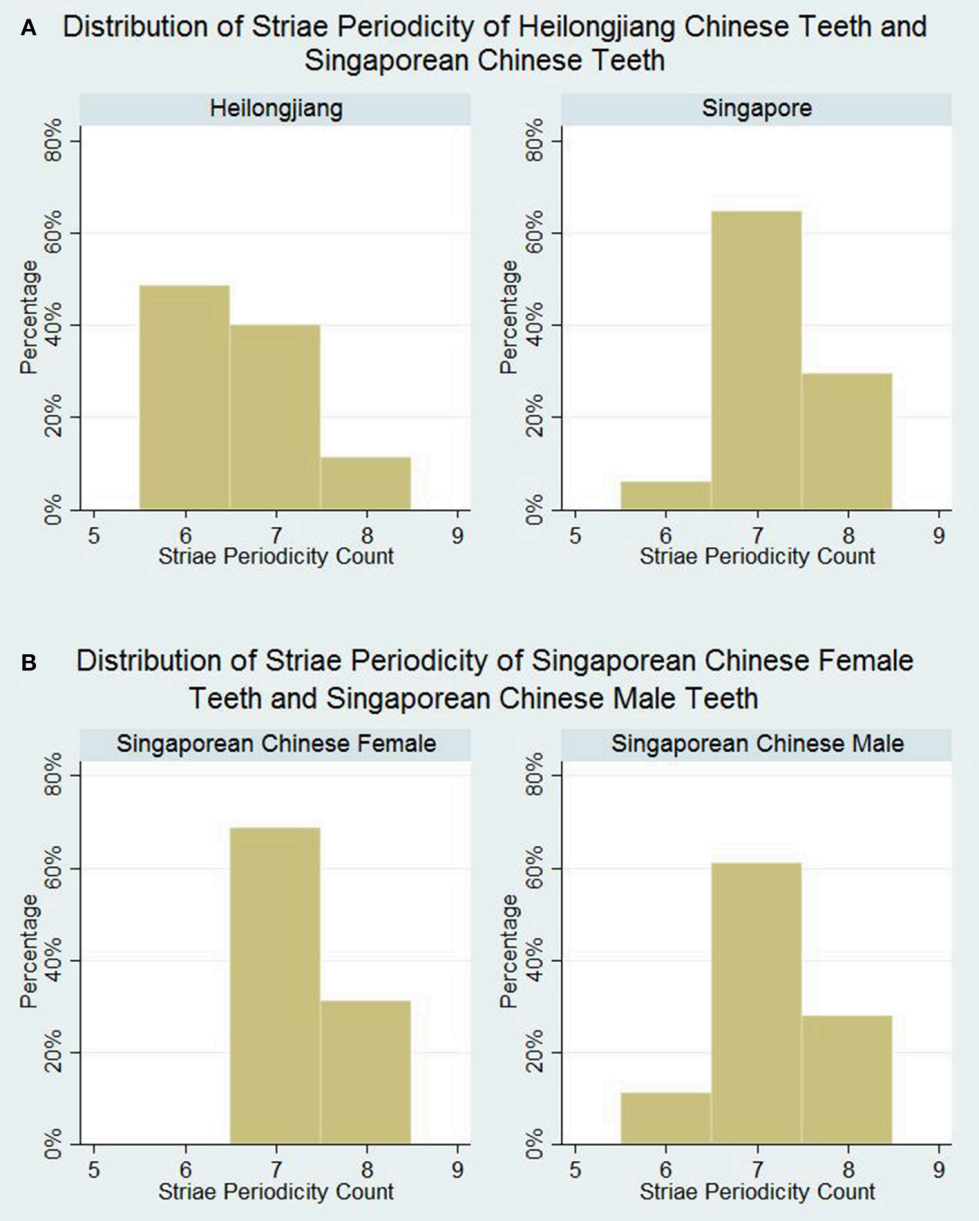
Distribution of striae periodicity. **(A)** Distribution of striae periodicity of Heilongjiang Chinese teeth and Singaporean Chinese teeth. **(B)** Distribution of striae periodicity of Singaporean Chinese female teeth and Singaporean Chinese male teeth.

According to the Kolmogorov-Smirnov test for normality, the median striae periodicities of the samples of teeth from both Heilongjiang and Singapore do not follow a normal distribution. Median striae periodicity counts were 7 for both Singapore Chinese group and Heilongjiang Chinese group. However, there is a statistically significant difference in the distribution of striae periodicity counts (*p* < 0.001). As seen in Figure 2A, Heilongjiang Chinese teeth tend to have lower striae periodicity counts as compared to Singaporean Chinese teeth. Close to half (49%) of the Heilongjiang Chinese group had striae periodicity counts of 6, while 94% of the Singapore Chinese group had striae periodicity counts of 7 or 8.

Median striae periodicity counts were reported at 7 for both Singaporean Chinese male teeth and Singaporean Chinese female teeth (Figure 2B). No statistically significant difference in striae periodicity counts was observed between the teeth from Singapore Chinese males and Singapore Chinese females (*p* = 0.511).

## Discussion

Our results suggest that there is a higher striae periodicity of teeth from Singapore than that from Heilongjiang. This is similar to the results published on the striae periodicity of teeth from different geographical locations, which revealed that South Africans have a higher mean periodicity than North Europeans and North Americans (Reid and Dean, 2006). While no clear explanations have been given for this difference in the existing literature, one plausible explanation is that people in different geographic locations experience different degrees of light exposure (Burgess, 2009). Assuming the amount of exposure to artificial light in both populations is similar, overall light exposure is higher for Singaporean Chinese than Heilongjiang Chinese due to shorter daytimes in winter for the latter. Given that the human suprachiasmatic nuclei synchronizes internal metabolic circadian rhythms to external light and dark cycles, shorter light hours may alter neuron firing patterns in the suprachiasmatic nuclei via the light sensitive retino-hypothalamic pathway (Brancaccio et al., 2014), resulting in a lower striae periodicity in Heilongjiang teeth. Study data have suggested that circadian clock genes regulate enamel secretion and mineralization by ameloblasts (Zheng et al., 2011), and amelogenin gene expression has been found to be two-fold lower during dark periods compared to light periods (Lacruz et al., 2012). Nevertheless, it is inconclusive if the difference identified in this study is biologically or clinically significant.

In 2012, Bromage et al. (2012) investigated another model of biologic rhythm—the Havers Halberg oscillation (HHO). HHO has been proposed to drive long term biologic rhythms that determine the regular periodicity of Striae of Retzius in enamel, as well as of bone lamella formation. It is generated in the hypothalamus, and regulates growth and body mass through pituitary secretions. The body mass and basal metabolic rates of primates have, in turn, been found to be significantly correlated with striae repeat interval (Bromage et al., 2012). HHO rhythms are said to have evolved in response to selection pressures. Applying the intra-specific HHO (Mahoney et al., 2016), the tropical warm climate of Singapore may favor a different oscillation of the HHO compared to that in the temperate climate of Heilongjiang, leading to higher basal metabolic rates and higher long term striae periodicities. Nevertheless, this argument may be potentially defective due to findings that basal metabolic rates either do not differ between temperate and “hot” climates (Ocobock, 2016), or are lower in tropical climates than temperate climates (Mason and Jacob, 1972).

Furthermore, Newman and Poole hypothesized that a Retzius line is formed when a free running endogenous circadian rhythm is most offset from a more precise 24-h exogenous circadian rhythm (Newman and Poole, 1993). It may be possible that environmental cues such as temperature and climate that vary with geographic location result in variations in the time at which the two cycles are most offset from each other, resulting in a difference in striae periodicity.

Our study showed no statistically significant gender difference in striae periodicity, which is echoed in the study by Schwartz et al. (2001). The lack of disparity between Singaporean Chinese male and female teeth in our study may be cautiously interpreted taking into consideration the following factors. First, the teeth selected in our study were mainly premolars and molars, rather than canines that are thought to be the most sexually dimorphic in terms of size and shape (Plavcan, 2001). Male canines are on average larger and heavier than female canines (Schwartz and Dean, 2005). Nevertheless, this reasoning may be disputed as striae periodicities in different tooth types (incisors, canines, premolars, molars) has been found to be equal in a single individual (FitzGerald, 1998; Reid et al., 1998; Mahoney, 2012). Second, some studies that reported different striae periodicity values between male and female genders did not set geographical location as a constant (FitzGerald, 1998). Since striae periodicity has been shown to vary according to geographical location in our study, the differing results in other studies may be attributed to confounders, such as geographic location. Third, our study is slightly underpowered (power = 78.6%) for the investigation of gender difference at an effect size of 1, based on a *post-hoc* power analysis. Nevertheless, the observed distributions of striae periodicity counts of males and females were similar (Figure 2B), suggesting that gender differences may not be clinically relevant.

For reliability testing, the ICC of 0.92 reflects excellent inter-observer reliability. The ICC for all three observers are between 0.5 and 0.75, indicating moderate intra-observer reliability (Cicchetti, 1994; Koo and Li, 2016). The difficulties in striae measurement, due to striae convergence at the tooth surface and the presence of intradian lines and laminations that confound the striae counting process (Smith et al., 2003), may have contributed to the minor differences in final and repeat striae counts by the same observer.

### Applications

The total cross-striation count after birth has been found to be highly consistent with those expected from the known ages (Risnes, 1998; Stavrianos et al., 2010). The knowledge of average striae periodicity values of Heilongjiang and Singaporean Chinese populations may thus allow us to make more accurate age estimations in forensic dentistry based on incompletely formed primary and permanent teeth.

The research also adds value in the academic field, particularly in the study of dental histology, by offering an insight into the influence of gender on striae periodicity. The study is one of the first of its kind that examines samples from Asian populations, and investigates the effect of geographical location on striae periodicity, with ethnicity kept constant. As such, this study serves as a stepping-stone for future studies.

### Limitations

Logistical limitations prevented the procurement of gender information for the Heilongjiang samples. Further studies with multivariate regression analyses could be conducted to investigate the relationship between demographic and biopsychosocial factors, and striae periodicity counts.

As this study only involved the use of Polarized Light Microscopy, data cannot be confirmed should there be errors due to instrument or measurement limitations. To further improve the study quality, alternative microscopy techniques such as laser confocal scanning microscopy may be explored (Antoine and Dean, 2009).

## Conclusion

Results show a statistically significant difference between striae periodicity values of Heilongjiang and Singaporean Chinese, but not between gender groups. Further studies with different instruments and methodologies may be required to identify other confounders of striae periodicity values.

## Author contributions

ST and CH were involved in the conception and design of the work, data acquisition, analysis, interpretation, and drafting and revision of the manuscript. YS was involved in data analysis and interpretation, and revising the manuscript. All authors are responsible for final approval of the version to be published and agree to be accountable for the content of the work.

### Conflict of interest statement

The authors declare that the research was conducted in the absence of any commercial or financial relationships that could be construed as a potential conflict of interest.
