# Supplementary material for: Difference in Striae Periodicity of Heilongjiang and Singaporean Chinese Teeth
Source: Front Physiol. 2017 Jun 29;8:442. doi: 10.3389/fphys.2017.00442 (PMC5489628; doi:10.3389/fphys.2017.00442)
Supplement: Supplementary file 1 [file Table1.DOCX]

Supplementary Material

Article Title

Sharon Hui Xuan Tan^1^, Chin-Ying Stephen Hsu^2*^

*** Correspondence:** Chin-Ying Stephen Hsu: denhsus@nus.edu.sg

# Supplementary Data

**Supplementary Table 1: Summary of Mean and Modal Striae Periodicities reported in literature**

| Study | Sample | Mean | Mode | Range |
| --- | --- | --- | --- | --- |
| Fitzgerald 1998 | 158 anterior teeth from Amerindians, Europeans, and Africans | 9.44 (Amerindians); 9.87 (Europeans); 9.87 (Africans); 9.71 (Overall) | 9.00 (Amerindians); 9.2 (Europeans); 10.00 (Africans); 9.20 (Overall) | 7.80-12.30 |
| Schwartz, Reid and Dean 2001 | 19 male, 9 female teeth from UK and South Africa | 8.37 (Males), 9.00 (Females) | 8.00 | 7.00-11.00 |
| Reid and Dean 2006 | 678 teeth from North Europeans, South Africans, North Americans (Caucasian, mixed), Medieval Danish | 7.69 (North European upper 2^nd^ molar) – 9.60 (South African upper central incisor) | 8 (posterior teeth)  9 (anterior teeth) | 6-12 |
| Smith 2007 | 420 molar teeth from 365 individuals from South Africa (n=121),  Northern England (n=83), North America (n=100) and medieval Denmark (n=61) | 8.6 (South Africa); 8.1 (North England); 7.9 (North American); 8.4 (Medieval Danish);  8.3 (Overall) | 8 (South Africa, North England, North America)  9 (Medieval Danish)  8 (Overall) | 6-12 (South Africa); 6-11 (North England); 7-9 (North America); 7-11 (Medieval Danish) |
| Reid & Ferrell 2006 | 49 mandibular canines from medieval Danish | 8.5 | 8 | 7-11 |
| Lacruz 2006 early Homo | 17 molars from Plio-Pleistocene hominins | 8 | 8 | 7-9 |
| Lacruz 2006 australopithecine |  | 7 | 7 | 7 |

# Supplementary Figures and Tables

**Supplementary Table 2:** **Striae Periodicity of Heilongjiang Chinese Teeth**

| SN | Sample 1 | | | Sample 2 | | | Overall Median |
| --- | --- | --- | --- | --- | --- | --- | --- |
|  | Observer A | Observer B | Observer C | Observer A | Observer B | Observer C |  |
| C1 | 8 | 8 | 8 | 7 | 7 | 8 | 8 |
| C2 | 8 | 8 | 8 | 8 | 9 | 8 | 8 |
| C3 | 8 | 8 | 8 | 7 | 7 | 8 | 8 |
| C4 | 7 | 7 | 7 | 8 | 7 | 7 | 7 |
| C5 | 7 | 7 | 7 | 6 | 6 | 7 | 7 |
| C6 | 7 | 7 | 7 | 7 | 7 | 7 | 7 |
| C7 | 7 | 7 | 7 | 8 | 7 | 8 | 7 |
| C8 | 6 | 6 | 6 | 6 | 6 | 6 | 6 |
| C9 | 7 | 7 | 7 | 7 | 7 | 7 | 7 |
| C10 | 7 | 6 | 7 | 7 | 6 | 7 | 7 |
| C11 | 6 | 6 | 7 | 6 | 6 | 7 | 6 |
| C12 | 7 | 7 | 7 | 7 | 7 | 7 | 7 |
| C13 | 7 | 7 | 7 | 6 | 6 | 7 | 7 |
| C14 | E | E | E | 6 | 6 | 6 | 6 |
| C15 | 6 | 6 | 6 | 6 | 6 | 7 | 6 |
| C16 | 6 | 6 | 6 | 6 | 6 | 6 | 6 |
| C17 | 7 | 7 | 7 | 7 | 7 | 8 | 7 |
| C18 | 7 | 6 | 7 | E | E | E | 7 |
| C19 | 6 | 6 | 7 | 6 | 6 | 7 | 6 |
| C20 | 6 | 6 | 6 | 6 | 6 | 6 | 6 |
| C21 | 8 | 8 | 8 | 8 | 8 | 8 | 8 |
| C22 | 7 | 6 | 7 | 6 | 6 | 6 | 6 |
| C23 | 7 | 7 | 7 | E | E | E | 7 |
| C24 | 7 | 7 | 6 | 6 | 6 | 6 | 6 |
| C25 | 6 | 6 | 6 | 6 | 6 | 6 | 6 |
| C26 | 6 | 6 | 6 | E | E | E | 6 |
| C27 | 6 | 6 | 6 | 6 | 6 | 6 | 6 |
| C28 | 6 | 6 | 6 | E | E | E | 6 |
| C29 | 6 | 6 | 6 | E | E | E | 6 |
| C30 | 6 | 6 | 6 | 6 | 6 | 6 | 6 |
| C31 | 6 | 6 | 6 | 6 | 6 | 7 | 6 |
| C32 | 6 | 6 | 7 | 7 | 7 | 7 | 7 |
| C33 | 7 | 7 | 7 | E | E | E | 7 |
| C34 | 6 | 6 | 6 | 6 | 7 | 6 | 6 |
| C35 | 6 | 6 | 7 | 7 | 7 | 7 | 7 |

**Supplementary Table 3: Striae Periodicity of Singaporean Chinese Teeth**

| SN^*^ | Sample 1 | | | Sample 2 | | | Overall Median |
| --- | --- | --- | --- | --- | --- | --- | --- |
|  | Observer A | Observer B | Observer C | Observer A | Observer B | Observer C |  |
| SF1 | 7 | 7 | 7 | 7 | 7 | 7 | 7 |
| SF2 | 7 | 7 | 7 | 7 | 7 | 7 | 7 |
| SF3 | 7 | 7 | 7 | 7 | 7 | 7 | 7 |
| SF4 | 6 | 7 | 7 | 7 | 7 | 7 | 7 |
| SF5 | 7 | 7 | 7 | 7 | 6 | 7 | 7 |
| SF6 | 7 | 7 | 7 | 7 | 7 | 7 | 7 |
| SF7 | 8 | 7 | 7 | 8 | 8 | 8 | 8 |
| SF8 | 7 | 7 | 7 | 8 | 8 | 7 | 7 |
| SF9 | 7 | 7 | 7 | 7 | 7 | 7 | 7 |
| SF10 | 7 | 7 | 8 | E | E | E | 7 |
| SF11 | 7 | 6 | 7 | 7 | 7 | 7 | 7 |
| SF12 | 7 | 7 | 7 | 7 | 8 | 7 | 7 |
| SF13 | 8 | 8 | 8 | E | E | E | 8 |
| SF14 | 8 | 7 | 8 | 8 | 8 | 8 | 8 |
| SF15 | 8 | 7 | 8 | 8 | 7 | 8 | 8 |
| SF16 | 8 | 8 | 8 | 8 | 9 | 9 | 8 |
| SM1 | 7 | 7 | 7 | 8 | 7 | 7 | 7 |
| SM2 | 8 | 8 | 8 | 8 | 8 | 8 | 8 |
| SM3 | 8 | 8 | 8 | E | E | E | 8 |
| SM4 | 8 | 7 | 7 | 7 | 7 | 7 | 7 |
| SM5 | 7 | 7 | 7 | 7 | 6 | 7 | 7 |
| SM6 | 8 | 8 | 8 | 8 | 7 | 7 | 8 |
| SM7 | 7 | 7 | 6 | 7 | 7 | 7 | 7 |
| SM8 | E | E | E | 6 | 6 | 6 | 6 |
| SM9 | 7 | 7 | 7 | 7 | 7 | 8 | 7 |
| SM10 | 7 | 8 | 7 | 7 | 7 | 7 | 7 |
| SM11 | 9 | 8 | 8 | 8 | 8 | 8 | 8 |
| SM12 | 8 | 8 | 8 | 8 | 8 | 9 | 8 |
| SM13 | 8 | 7 | 7 | 7 | 7 | 7 | 7 |
| SM14 | 6 | 7 | 7 | 6 | 6 | 6 | 6 |
| SM15 | 7 | 7 | 7 | 7 | 7 | 7 | 7 |
| SM16 | 7 | 7 | 7 | 7 | 7 | 7 | 7 |
| SM17 | 7 | 7 | 7 | 7 | 7 | 7 | 7 |
| SM18 | 7 | 7 | 7 | 7 | 7 | 7 | 7 |

**Legend:**

**E: Excluded Sections (e.g. striae uncountable, damaged sections)**

*** SF refers to Singaporean Chinese Females, SM refers to Singaporean Chinese Males**
